# Forecasting Distributional Responses of Limber Pine to Climate Change at Management-Relevant Scales in Rocky Mountain National Park

**DOI:** 10.1371/journal.pone.0083163

**Published:** 2013-12-31

**Authors:** William B. Monahan, Tammy Cook, Forrest Melton, Jeff Connor, Ben Bobowski

**Affiliations:** 1 Inventory and Monitoring Division, National Park Service, Fort Collins, Colorado, United States of America; 2 Biological Resource Management Division, National Park Service, Fort Collins, Colorado, United States of America; 3 California State University Monterey Bay, Seaside, California, United States of America; 4 Cooperative for Research in Earth Science and Technology, NASA Ames Research Center, Moffett Field, California, United States of America; 5 Rocky Mountain National Park, Estes Park, Colorado, United States of America; University of Saskatchewan, Canada

## Abstract

Resource managers at parks and other protected areas are increasingly expected to factor climate change explicitly into their decision making frameworks. However, most protected areas are small relative to the geographic ranges of species being managed, so forecasts need to consider local adaptation and community dynamics that are correlated with climate and affect distributions inside protected area boundaries. Additionally, niche theory suggests that species' physiological capacities to respond to climate change may be underestimated when forecasts fail to consider the full breadth of climates occupied by the species rangewide. Here, using correlative species distribution models that contrast estimates of climatic sensitivity inferred from the two spatial extents, we quantify the response of limber pine (*Pinus flexilis*) to climate change in Rocky Mountain National Park (Colorado, USA). Models are trained locally within the park where limber pine is the community dominant tree species, a distinct structural-compositional vegetation class of interest to managers, and also rangewide, as suggested by niche theory. Model forecasts through 2100 under two representative concentration pathways (RCP 4.5 and 8.5 W/m^2^) show that the distribution of limber pine in the park is expected to move upslope in elevation, but changes in total and core patch area remain highly uncertain. Most of this uncertainty is biological, as magnitudes of projected change are considerably more variable between the two spatial extents used in model training than they are between RCPs, and novel future climates only affect local model predictions associated with RCP 8.5 after 2091. Combined, these results illustrate the importance of accounting for unknowns in species' climatic sensitivities when forecasting distributional scenarios that are used to inform management decisions. We discuss how our results for limber pine may be interpreted in the context of climate change vulnerability and used to help guide adaptive management.

## Introduction

Protected areas are a primary means to conserving biodiversity, but species afforded local protection are often distributed considerably beyond administrative boundaries [1]–[3]. This challenge to place-based conservation and management is further exacerbated by climate and other forms of environmental change that operate over broad spatial scales [4]–[6]. In the US, the Department of the Interior recognized the need for new, landscape-level approaches to resource management and responded by establishing a system of Landscape Conservation Cooperatives (LCCs) [7]. LCCs are intended to facilitate the co-management of shared species and other resources across jurisdictional boundaries, but management decisions are still ultimately made and enacted by individual management units belonging to agencies and institutions with sometimes very different missions [8], [9]. Given these challenges – and new opportunities afforded by LCCs – managers of protected areas require a detailed understanding of how their key, defining species will respond at multiple scales to ongoing and future environmental change. Here, we forecast possible distributional responses of limber pine (*Pinus flexilis*) to climate change in Rocky Mountain National Park (Colorado, USA) using species distribution models parameterized at management-relevant scales.

Limber pine is a species of white pine (subgenus *Strobus*) in North America that influences three major ecosystem processes important to managers: (i) post-fire succession [10], (ii) food provisioning for wildlife [11]–[13], and (iii) snow accumulation and retention [14], [15]. Limber pine is considered a species of management concern [16], [17], primarily due to recent and widespread tree mortality events caused by native mountain pine beetle [18], [19] and invasive white pine blister rust [20], [21]. Climate change is anticipated to interact with and exacerbate these threats in complex ways [22], [23], and it may further shorten the fire return interval in ways that either favor (increased disturbance) or disfavor (unable to reach reproductive age) limber pine, but at a more proximate and tractable level for forecasting it is also expected to exert direct effects on particular dominance and age classes [24]–[26].

Focusing on these direct effects, limber pine has the broadest elevational range of any tree species in Colorado, ranging from 1600 m on the plains to tree-line at 3300 m [27]. The species also occurs throughout most western states [28]. Such broad geographical and elevational limits to distribution raise important and unanswered questions about the spatial extent at which climatic sensitivity (i.e., a species' ability to tolerate a change in climate [29]) should be inferred from limber pine occurrence data and used to model distributional responses to climate change in individual parks and other protected areas. On the one hand, if the traits governing distribution are truly conserved at the species level, inferring climatic sensitivity from limber pine occurrences in and near Rocky Mountain National Park will underestimate the ability of local populations to respond to future change. On the other, if local adaptation or community dynamics largely determine limber pine distribution in and near the park, inferring sensitivity rangewide will overestimate the ability of local populations to respond to future change. Because we do not know the precise mechanisms that govern distributional limits in limber pine, a practical compromise approach for developing forecasts useful to managers is to parameterize models using a range of plausible climatic sensitivities that are inferred from multiple spatial extents [30].

Our analyses bracket local and rangewide spatial extents to evaluate how the size, position, and shape of limber pine distribution in Rocky Mountain National Park may change throughout the 21^st^ century in response to climate change. We address two questions important to park managers: (i) how long will areas within the current limber pine distribution in the park remain climatically suitable, and (ii) when and where will areas outside this current distribution become more climatically suitable than present? We evaluate two representative concentration pathways (RCP 4.5 and 8.5), designed to capture uncertainty in future greenhouse gas (GHG) emission rates, as expressed by their equivalent radiative forcings in the year 2100 in watts per square meter [31], and ask whether future climate uncertainty or spatial extent has a greater effect on our confidence in distributional responses of limber pine to climate change. We conclude with a discussion of how results may be used to inform assessments of climate change vulnerability [29] and guide possible management strategies.

## Materials and Methods

### Species Occurrence Data

Limber pine occurrences were obtained from two data sources that differ in spatial extent: (i) rangewide from the Whitebark and Limber Pine Information System (WLIS) [32], and (ii) locally within Rocky Mountain National Park from the National Park Service (NPS) Vegetation Inventory Program (VIP) [33]. WLIS is a database that compiles from the scientific literature all limber pine observations throughout the species' geographic range. While WLIS is not a single comprehensive survey across the entire geographic distribution of limber pine, the occurrence data compiled by WLIS are broadly distributed and include marginal areas of the species' range with respect to latitude, longitude, and elevation. Approximately 93% of the limber pine range occurs in the US [28] – the maximum geographic extent of our climate data (described below) – and a total of 260 georeferenced WLIS limber pine point localities from the US were considered in the rangewide analysis. Although 14 localities from Canada were excluded, these observations are within 24 to 248 km (mean 68 km) of the northernmost localities in the US (US localities span approximately 1500 km); Canadian localities are also encompassed both longitudinally and elevationally by the US localities. Hence, climates of Canadian localities are thought to be reasonably approximated by the US localities. After removing duplicate point observations at the resolution of the climate data (30 arc-seconds or ∼800 m; see below) – to reduce the effects of geographic biases in sampling – a total of 237 spatially unique point localities were used to train the rangewide limber pine models.

VIP data were collected within 1731 km^2^, including Rocky Mountain National Park plus a 1.6 km buffer to the north, west and south, and a 6.4 km buffer to the east, which included an extensive urban interface. In total, 632 vegetation plots were sampled in 2002, where plot size in habitats containing limber pine was 400 m^2^. Limber pine was dominant in two woodland National Vegetation Classification (NVC) types: Limber Pine/Kinnikinnick Woodland (CEGL000802) and Limber Pine/Common Juniper Woodland (CEGL000807). In the plots, limber pine also commonly co-occurred with Subalpine Fir-Engelmann Spruce (CEGL000986) and Subalpine Fir-Krummholz Shrubland (CEGL000985), but when mapped as a dominant class using aerial imagery it was limited to CEGL000802 and CEGL000807, which encompassed 309 contiguous polygons for a total of 26.81 km^2^ (mean: 0.087 km^2^; 95% CI: 0.004–0.309 km^2^; estimated accuracy 82.4–96.6%). From these polygons we computed the percentage area in each ∼800×800 m climate grid cell (388 grid cells encompassing limber pine; 2646 grid cells encompassing the extent of the park vegetation map) and used these percentages to assign weights to point localities used to train the local limber pine models. Assigning weights in this fashion assumes that grid cells with a higher percentage of limber pine are climatically more suitable or favorable than grid cells with lower percentages, due to fine-scale topographic heterogeneity and the distribution of limber pine suitable land facets that influence microclimates.

### Climate Data

Contemporary climate data were obtained from the parameter regression on independent slopes model, PRISM [34]. We selected PRISM 1981–2010 normals (30 arc-seconds, ∼800 m spatial resolution) for analysis; this 30-year period provided an estimate of contemporary climate (i.e., smoothing over interannual variability in weather) that was also temporally concomitant with the vegetation inventory. We obtained data for mean minimum monthly temperature, mean maximum monthly temperature, and total monthly precipitation.

Future climate projections were obtained from the NASA Earth Exchange (NEX) Downscaled Climate Projections (DCP) for the conterminous US (NEX-DCP30) [35]. The NEX-DCP30 dataset includes more than 100 climate simulations conducted as part of the Coupled Model Intercomparison Project Phase 5 (CMIP5), downscaled to 30 arc-seconds using the Bias-Correction Spatial Disaggregation (BCSD) approach [36]–[38]. Monthly PRISM data from 1950 through 2005 were used to produce the NEX-DCP30 dataset, as a training reference in BCSD. From this dataset, we selected for analysis a yearly sequence of 30-year normals (2006–2035, 2007–2036 … 2071–2100) designed to encompass both near- and long-term management considerations. We also selected two representative concentration pathways (RCP 4.5 and 8.5) designed to reasonably bracket the lower and upper extremes of modeled changes in temperature [31]. Individual model simulations from a total of 31 general circulation models (GCMs) were downscaled under both RCP 4.5 and 8.5: ACCESS1.0, BCC-CSM1.1, BCC-CSM1.1(m), BNU-ESM, CanESM2, CCSM4, CESM1(BGC), CESM1(CAM5), CMCC(CM), CNRM-CM5, CSIRO-Mk3.6.0, FGOALS-g2, FIO-ESM, GFDL-CM3, GFDL-ESM2G, GFDL-ESM2M, GISS-E2-R, HadGEM2-AO, HadGEM2-CC, HadGEM2-ES, INM-CM4, ISPL-CM5A-LR, ISPL-CM5A-MR, ISPL-CM5B-LR, MIROC-ESM, MIROC-ESM-CHEM, MIROC5, MPI-ESM-LR, MPI-ESM-MR, MRI-CGCM3, NorESM1-M. From these, we used the ensemble averages for mean minimum monthly temperature, mean maximum monthly temperature, and total monthly precipitation to provide overall consensus estimates of future climate.

The gridded contemporary and future monthly climate variables (30-year normals) were used to calculate a series of more biologically meaningful variables, termed bioclimatic variables. A total of 19 bioclimatic variables were considered in developing the limber pine distribution models [39] (Table 1). Future climate data used in projecting the limber pine models were first calculated as delta surfaces from the NEX-DCP30 1981–2010 baseline; we then added these deltas to the PRISM data used in model training so as to ensure that predicted changes in limber pine distribution were not due to any pixel-level artifacts between the training and projection climate data.

**Table 1:** Bioclimatic variables considered in the distribution models of limber pine.

Code	Name
Bio 1	Annual mean temperature
Bio 2	Mean diurnal range (mean of monthly (max temp – min temp))
Bio 3	Isothermality (Bio 2/Bio 7)
Bio 4	Temperature seasonality (standard deviation)
Bio 5	Maximum temperature of the warmest month
Bio 6	Minimum temperature of the coldest month
Bio 7	Temperature annual range (Bio 5–Bio 6)
Bio 8	Mean temperature of the wettest quarter
Bio 9	Mean temperature of the driest quarter
Bio 10	Mean temperature of the warmest quarter
Bio 11	Mean temperature of the coldest quarter
Bio 12	Annual precipitation
Bio 13	Precipitation of the wettest month
Bio 14	Precipitation of the driest month
Bio 15	Precipitation seasonality (coefficient of variation)
Bio 16	Precipitation of the wettest quarter
Bio 17	Precipitation of the driest quarter
Bio 18	Precipitation of the warmest quarter
Bio 19	Precipitation of the coldest quarter

### Species Distribution Models

Distribution models of limber pine were developed using MaxEnt [40], version 3.3.3k, although analyses could utilize any of the common presence-only or presence-absence modeling methods [41], as well as more complex dynamic range simulation methods that rely on niche theory [42], [43]. Models were developed at two spatial extents: rangewide (conterminous US) and local (Rocky Mountain National Park). Importantly, this comparison holds resolution constant (∼800 m) while varying the size of the geographic domain. Seo et al. [44] show that – holding the spatial extent constant – this grid cell size generally results in higher measures of model performance, compared to models developed at coarser spatial resolutions.

The geographic domain associated with the rangewide model was delineated by all Commission for Environmental Cooperation Level III ecoregions [45] containing the limber pine range [28]. The geographic domain associated with the local model was delineated by the geographic extent of the VIP map. Pseudo-absence data (i.e., background points) were thus constrained to each of these two spatial extents, and we generally accepted default MaxEnt settings as tested and recommended by Phillips & Dudík [46], but we did not render occurrence data spatially unique because: (i) our rangewide point occurrence training data had already been rendered spatially unique at the resolution of the climate data, and (ii) we used the percentage of limber pine area in each ∼800×800 m climate pixel to weight point occurrence training data, thus allowing climate pixels with higher cover percentages to be more influential in training the model, under the premise that they also have more suitable microclimates driven by fine-scale topography.

The 19 bioclimatic variables used to parameterize the rangewide and local models are, to varying degrees, correlated. This knowledge, coupled with a desire to reduce unnecessary variable interactions to simplify models for purposes of projecting to future climates, prompted us to test among a series of 6 competing models with different variables: (i) all 19 bioclimatic variables (Bio 1–19), (ii) annual (Bio 1–4, 7, 12, 15), (iii) monthly (Bio 2–7, 13–15), (iv) quarterly (Bio 2–4, 7–11, 15–19), (v) temperature (Bio 1–11), and (vi) precipitation (Bio 12–19). At the local extent, we produced MaxEnt models for each combination of variables and then used Environmental Niche Modeling (ENM) Tools [47], [48], version 1.3, to calculate Akaike Information Criterion, with correction for finite sample size (AICc), and ΔAICc, where models yielding ΔAICc≤2 were considered equally top performing [49]. Bioclimatic variables associated with the top performing local model were used to parameterize the rangewide model, thus controlling for this factor in the comparisons.

Following model training and comparison, we used MaxEnt to develop logistic probability projections for the top performing models. All projections were restricted in spatial extent to the VIP map. Hence, although models were trained at two spatial extents, and the rangewide model was developed using occurrence data collected throughout the US, both local and rangewide map predictions were evaluated for areas inside and proximate to the park (i.e., the spatial scale of influence for park managers). Temporally, projections were developed for the training period (1981–2010) and the full yearly sequence of future 30-year normals (2006–2035, 2007–2036 … 2071–2100), considered under both RCP 4.5 and 8.5. We evaluated the complete time series in order to expose any rapid, non-linear changes in the distributional metrics (below).

### Statistical Analysis

Our analyses of the size, position, and shape of limber pine distribution over time necessarily required us to render the logistic projections binary. We achieved this using the MaxEnt-provided logistic threshold equating the entropy of thresholded and original distributions, calculated separately for the rangewide (0.159) and local (0.174) models. To further test whether the dominant limber pine class in the park was randomly vs. non-randomly predicted by the rangewide model, we queried the rangewide predictions against all and dominant-only VIP grid cells and compared the mean and confidence interval (CI) of logistic probabilities; evidence of a non-random distribution of dominant logistic probabilities was assessed based on differences between the means and the extent to which CI's were non-overlapping. The two statistical distributions were considerably different, suggesting that dominant limber pine was non-randomly predicted by the rangewide model, so we calculated a new rangewide threshold (0.434) that yielded the same sensitivity (true positive rate) as the dominant model trained at the local extent.

For each model×threshold combination (local 0.174, rangewide 0.159, rangewide 0.434), we calculated the area (size), mean elevation (position), and core patch index (shape) of each projection; these metrics in different ways affect the ability of limber pine to influence key ecosystem processes important to managers. Elevations were obtained from the National Elevation Dataset [50]; digital elevation model (DEM) data were obtained originally at 30 m resolution and bilinearly resampled to match the resolution of model predictions. The core patch index is a measure of the degree to which the observed core patch area approaches an idealized patch area, where core area is maximized while controlling for total area. It is calculated as *A_c_*/(√A−2)^2^, where *A_c_* is the total area of core patch, estimated here with an edge width of 1 pixel or grid cell, and *A* is the total area of the predicted limber pine distribution (i.e., including edge pixels).

We then quantified over time the extent to which limber pine distribution moved or shifted outside the current observed range of the species, as documented and recorded by the vegetation inventory. This was achieved by separately intersecting and taking the union of the binary projections with the grid cells containing dominant limber pine polygons. For each projection, we calculated: (i) area shift as the total area intersect divided by the total area union, (ii) elevation shift as the total elevational range intersect divided by the total elevational range union, and (iii) core patch area shift as the core patch area intersect divided by the core patch area union; all ratios were multiplied by 100 and expressed as percentages.

Finally, we used an additional feature offered by MaxEnt to understand when and where model projections were being extrapolated beyond the training space and into novel climates. The multivariate environmental similarity surface (MESS) measures, for each pixel, the degree to which training and projection environments are the same, where negative values identify dissimilar pixel pairs [51], [52]. Negative MESS values are important in management considerations because they identify areas of extreme uncertainty. For each training spatial extent (rangewide and local) and each RCP (4.5 and 8.5), we intersected each binary projection with its associated MESS and calculated the MESS mean across binary grid cells to estimate if, or when, in the future model projections turned negative.

## Results

At the local extent, the full model considering all 19 bioclimatic variables yielded the lowest estimates for AICc (Table 2). Models based on the quarterly variables had the second lowest estimates of AICc, and those based on monthlies had the third lowest, but neither of these yielded acceptably low values for ΔAICc (Table 2). Hence, all rangewide and local analyses were based on full models.

**Table 2:** Akaike Information Criterion, with finite sample size correction (AICc), for distribution models of limber pine trained at the local extent using six different combinations of bioclimatic variables.

Bioclimatic variables	Log Likelihood	Parameters	Sample Size	AICc score	ΔAICc
All	−25503.98	225	4297	51482.94	0.00
Annual	−26874.08	138	4297	54033.39	2550.45
Month	−26427.78	157	4297	53181.55	1698.61
Quarter	−25842.27	196	4297	52095.37	612.43
Temperature	−26725.58	164	4297	53792.27	2309.33
Precipitation	−26992.15	161	4297	54318.91	2835.97

**Figure 1:**
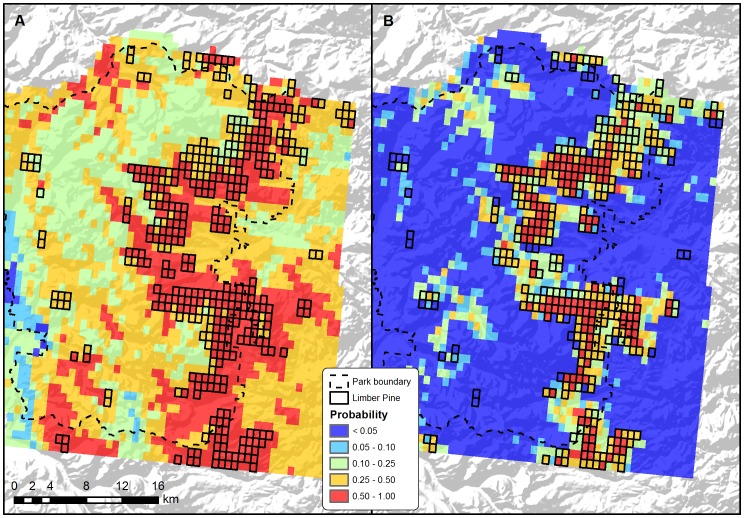
Predicted probability of current (1981–2010) limber pine occurrence in Rocky Mountain National Park. MaxEnt models trained at two spatial extents: A) rangewide, and B) at the local extent considering only those areas where limber pine is the dominant vegetation class.

Current predicted probabilities of limber pine occurrence in and near Rocky Mountain National Park differed dramatically between the models trained at the rangewide and local extents, especially throughout areas where limber pine is not the dominant vegetation class (Figure 1). In these areas, the rangewide model predicted occurrence at relatively high probability (0.1–0.5), whereas the local model predicted occurrence at extremely low probability (<0.05). Within areas where limber pine is the dominant vegetation class, both the rangewide and local models predicted occurrence at high probability (0.5–1.0, Figure 1). This correspondence suggests that, even at a rangewide extent, dominant limber pine is non-randomly distributed in climatic niche space. At the local extent of projection, this pattern is indeed confirmed by the non-random association of logistic probabilities for dominant-only grid cells (mean: 0.540, 95% CI: 0.258–0.716) vs. all grid cells (mean: 0.378, 95% CI: 0.122–0.678). Despite overlapping CIs, the differences are quite pronounced when compared to the logistic probabilities for the local model of dominant limber pine (mean: 0.384, 95% CI: 0.021–0.763), which has a relatively low mean probability due to some marginal grid cells receiving low values (Figure 1B).

**Figure 2:**
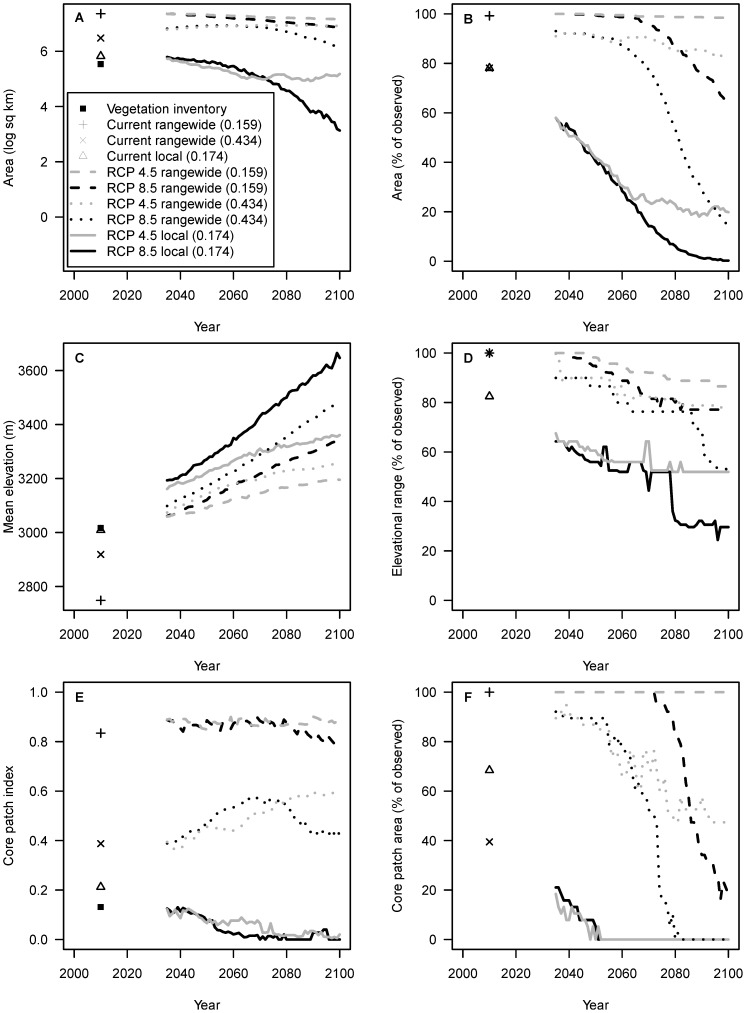
Future projections (2035–2100) of the distribution of limber pine in Rocky Mountain National Park. MaxEnt models trained at two spatial extents (rangewide and local) and projected under two future climate scenarios reflecting different GHG concentration pathways (RCP 4.5 and 8.5). Distributional summaries calculated from probability of occurrence maps rendered binary by the model thresholds reported in parentheses: A) total area; B) percentage area, within current observed area where limber pine is dominant; C) mean elevation; D) percentage elevational range, within current observed elevational range where limber pine is dominant; E) core patch index, and F) percentage core area, within current observed core area where limber pine is dominant.

Projecting the distribution of limber pine in the park through 2100, uncertainty surrounding the appropriate spatial extent for training the models is greater than the uncertainty in future climate conditions associated with the use of different RCPs (Figure 2). However, even in the face of these major sources of uncertainty, one pattern is consistent. Under both spatial extents and both RCPs, the distribution of limber pine in the park is projected to increase in elevation (Figure 2C). Projections for changes in area (Figure 2A) and patch fragmentation (Figure 2E), however, are highly variable and result in trends that occasionally even differ in direction.

Relating these projected changes to the current distribution of dominant limber pine in the park, the spatial extent of model training has a substantial effect on the question of whether, and when, to manage for stasis versus change (Figure 2). According to the model trained at the local extent, the percentage of total area (Figure 2B) and elevational range (Figure 2D) decreases to approximately 60% of the current observed distribution by 2035, while core patch area (Figure 2F) decreases to approximately 20%. By 2100, the RCPs have diverged, and these same measures decrease to approximately 50% (elevational range, RCP 4.5) to 0% (total area, RCP 8.5; core patch area, both RCPs). Meanwhile, according to the rangewide model, the measures decrease to a low of 90% of the current observed distribution by 2035, and by 2100 range anywhere from 100% (total and core patch area, RCP 4.5) to 0% (core patch area, RCP 8.5). If we restrict the rangewide model to dominant limber pine predictions, then the measures decrease approximately 85–0% by 2100. This restriction is reasonable since dominant limber pine in the rangewide model is predicted non-randomly throughout the park (Figure 1A). Thus, under a scenario that considers this thresholding of the rangewide model, plus the local model, the distribution of limber pine in the park is expected to move upslope and outside of its current elevational range during the 21^st^ century. Although both the magnitude and rate of change are uncertain (Figure 2D), a net effect is that dominant limber pine will also shift into new areas (Figure 2B, F).

Finally, novel future climates did not constitute a major source of uncertainty in the projections. Focusing on areas within the park where limber pine was predicted to occur, according to the different thresholds, mean MESS values from 2035 through 2100 were greater than zero under both spatial extents and RCPs, except for models trained at the local extent and projected to RCP 8.5. Under this model and scenario, mean MESS values turned negative in the year 2091, indicating that projections from this year forward should be interpreted with caution (Figure 2).

## Discussion

A distinct advantage of the species distribution models developed here is that they consider uncertainty in the spatial extent at which climatic sensitivities should be inferred and used to project local responses to climate change in individual parks and other protected areas. When applied to limber pine in Rocky Mountain National Park, results show: (i) the distribution is consistently predicted across models to move upslope in elevation and into new areas, and (ii) magnitudes and rates of change are more uncertain due to questions surrounding the appropriate spatial extent for inferring climatic sensitivity than they are to either future climate scenario (RCP 4.5 or 8.5) or the existence of novel future climates (MESS values). Combined, these results suggest that reducing uncertainty in the correlative model of limber pine distribution is presently more dependent on improving our integration of the species' geographical ecology than on refining our predictions of future environments.

Species often vary by age in their sensitivity to climate [53], [54]. In our analysis of limber pine in Rocky Mountain National Park, we had reason to believe that age classes were mixed within the dominant class, and most importantly, included seedlings and young trees that were recruited during the period of model training (1981–2010). Although older (200–300 year old) limber pine can succumb to drought stress [24], seedlings are an especially climate-sensitive life-stage [25]. In Rocky Mountain National Park, limber pine seedlings were found in 57% of plots surveyed and throughout all five major conifer vegetation types [55]. Hence, recruitment in the park appears to have been ongoing during the 1981 to 2010 period in which climatic sensitivity was inferred, suggesting that – despite the long-lived nature of limber pine at higher elevations [56] – it is reasonable to train a distribution model in contemporary time. An alternative approach would have been to reconstruct past climatic conditions of trees or stands based on their age [24], [57], but this was not possible due to a lack of available dendrochronological data at the necessary spatial extents. Importantly, extension of these analyses to other species should similarly consider both potential dominance and age effects on the forecasts, ensuring that any bias in either factor is accounted for in model interpretation. This is especially important given that disturbance history may not only impact community dominance, but also age class distributions, thus potentially causing age class disequilibrium with respect to climate, which in turn biases climate sensitivities inferred from occurrence data.

Our focus on areas where limber pine is the community dominant tree species was motivated by the fact that this structural-compositional vegetation class is relevant to park management because of the ecosystem processes it mediates [10]–[15]. The local model was trained on the dominant class while the rangewide model was indiscriminate with respect to dominance. This difference in dominance between the two models was intentional in that it allowed us to bracket the range of climatic sensitivities inferred from the observed species-climate associations. The low to moderate probabilities of occurrence predicted by the rangewide model throughout most areas of the park are plausible because limber pine is outcompeted by more shade-tolerant and rapidly maturing conifers [58]–[60], but exists rather pervasively as a minor component of other structural-compositional vegetation classes [33], [55]. Furthermore, the rangewide model predicted dominant limber pine almost exclusively at high probabilities, suggesting that predictions associated with this dominance class scale geographically and thus offer insights into future distributional responses that are independent of the model trained at the local extent. For managers seeking to integrate forecasts of species' distributional responses to environmental change into management scenarios, the benefits of this are two-fold: (i) when inferring environmental sensitivities from species' occurrences, it reasonably brackets plausible estimates of this important source of biological uncertainty, and (ii) when needing to look beyond protected area boundaries (e.g., to broader LCC-partnerships), it provides an opportunity to hierarchically evaluate distributional responses of particular management classes or categories at multiple spatial extents.

Despite opportunities to reduce uncertainty in this fashion in the present analysis, it is noteworthy that elevation emerged as the only distributional metric where predictions were consistent enough across spatial extents and climate scenarios to draw conclusions useful to park managers. This finding underscores the importance of understanding the scales at which species or taxa are in states of distributional equilibrium with respect to the environment, and how such uncertainty can have a dramatic effect on the utility of forecasts made from correlative models. In the case of limber pine, more certain predictions of total and core areas were desirable because these distributional metrics are believed to relate strongly to the potential for establishment in new areas [59], including especially those predicted at higher elevations beyond the current upper elevational limit of the species in the park. In an assessment of climate change vulnerability, such insights are valuable for quantifying the adaptive capacity of limber pine to colonize new environments and areas. However, important insights into adaptive capacity may also be gleaned from local field studies confirming the climatic changes, soils, and land facets needed for upslope establishment in areas that are currently above tree-line [25], [26], [61]. These studies collectively suggest that limber pine seedling establishment is not precluded by alpine soils and microenvironments, but also that moisture stress will impose major limits on upslope recruitment. Under the most extreme climate scenario considered in the present analysis (RCP 8.5), temperatures are projected to increase in the park, but so are annual and most seasonal measures of precipitation (except for Bio 14 (stable) and Bio 18 (decreasing); see also [62]). How exactly these translate to soil moisture content and seedling moisture stress – especially given interactions with nurse objects that further influence seedling microenvironments [63] – merits further study, but mechanistically, the increasing precipitation seen in the ensemble mean of the climate forecasts suggests the possibility that soil moisture conditions will remain – or perhaps become more – favorable. Soils are not presumed to be limiting because soil classes associated with limber pine inside the park also exist at higher elevations in areas that are presently alpine [64]. Of further note are transplantations made at Niwot Ridge, Colorado – immediately south of Rocky Mountain National Park – that show how limber pine seedlings can maintain key physiological functions at elevations above 3500 m [25]. This elevation is higher than all forecasted mean elevations for the year 2100, except for the most extreme forecasts captured by the local model projected to RCP 8.5, which, due to novel climates, are uncertain. Hence, projected future upslope movements of limber pine above tree-line are plausible given what is known about the high-elevation biology of the species.

**Figure 3:**
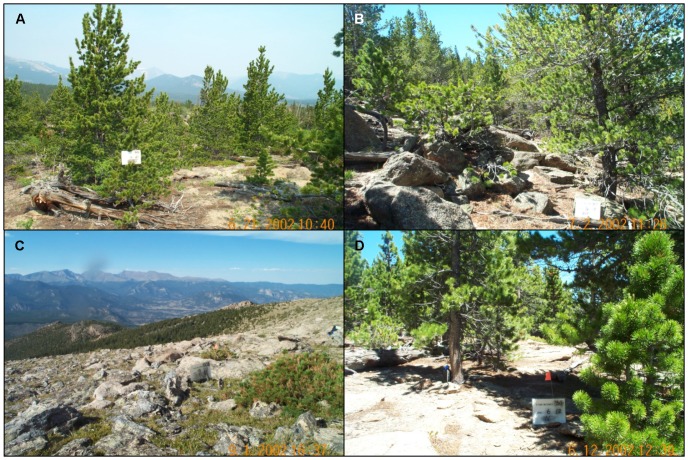
Examples of field plots containing limber pine in Rocky Mountain National Park, according to photos collected as part of the vegetation inventory[33]. A) Plot number 318, representative of the subalpine (dominant) limber pine map class. B) Plot number 303, representative of the subalpine (dominant) limber pine map class. C) Plot number 506, representative of the herbaceous upland alpine fellfield map class. D) Plot number 311, representative of the lodgepole pine – low elevation <9500 ft map class.

Future increase in the mean elevation of dominant limber pine in the park is further mediated by a concomitant increase in the lower elevational limit, as evident by a decrease in distributional area. Importantly, this does not equate to a loss of limber pine per se, but rather a loss of dominance status from lower elevations. Furthermore, the loss of dominant limber pine at lower elevations in the park is not explained by sampling constraints preventing immigration because even the rangewide model under RCP 8.5 predicted loss. Mechanistically, such loss is facilitated by succession and limber pine gradually becoming out-competed by more shade-tolerant and rapidly maturing conifers (Douglas-fir, *Pseudotsuga menziesii*; Engelmann spruce, *Picea engelmannii*; lodgepole pine, *Pinus contorta*; subalpine fir, *Abies lasiocarpa*). According to the vegetation inventory [33], some of this succession at low and high elevations may already be underway. At higher elevations, limber pine occurs in multiple strata of three of the alpine vegetation classes (herbaceous upland alpine >9600 ft, herbaceous upland alpine fellfield, shrub upland alpine). At lower elevations, but within the dominant limber pine vegetation class, all plots and strata containing limber pine also contain one or more of the shade-tolerant competitors (Douglas-fir, Engelmann spruce, lodgepole pine, subalpine fir), and in a subset of these the percentage cover of the competitor species combined represents 50–100% of the percentage cover reported for limber pine. Field photos from limber pine plots that span the compositional gradient further show what successional dynamics might look like on the ground (Figure 3). Combined, these observations from the vegetation inventory suggest the possibility that limber pine is already in the process of moving upslope, and that projected distributional increases in elevation from 2035 to 2100 are an acceleration of ongoing successional dynamics.

If the projected upslope movements occur, there may be some future need to manage limber pine for change, yet, from a management standpoint, it is useful to place both the magnitude and direction of elevational change in a longer term paleoclimatic perspective. In the early Holocene, limber pine was more common throughout lower elevations (<2500 m) than it is today [65], and many of the now isolated populations are believed to be remnants left behind by recent range retraction from lower elevations [66]. In Colorado, compared to present day, upper tree-line was generally higher in elevation from the early to middle-late Holocene, and generally at comparable or lower elevation over most of the last 3500 years [67]. More recently, since the end of the Little Ice Age (c. 1890), upper tree-lines have been re-advancing in elevation [61], [67], [68]. Combined, these past changes in elevation illustrate the degree to which tree-line has been in a state of flux, and suggest perhaps that some of the upslope movement predicted in the park throughout the 21^st^ century will constitute a re-colonization of alpine habitats by subalpine species. However, it is important to note that most of the paleoclimatic changes in tree-line elevation [67] are smaller in magnitude than those forecasted here, especially for the more extreme model projections. Hence, undoubtedly some of the late 21^st^ century projections reflect elevational changes that – should they occur – exceed a historical range of variability.

Given the elevational changes forecasted for limber pine in Rocky Mountain National Park, what might be some reasonable and feasible next steps to consider in an adaptive management context? One relates to monitoring and a test of the model prediction that limber pine is moving upslope. Even looking to the year 2035, the magnitudes of elevational change are sufficiently large that one would expect some limber pine recruitment to already be occurring in the lower alpine. The NPS Rocky Mountain Inventory and Monitoring Network has implemented an alpine vegetation monitoring protocol [69]. By c. 2090 under RCP 8.5, climates are projected to favor limber pine establishment in two of the four alpine monitoring sites [70], and forecasts presented here could be used to identify and prioritize additional sites to monitor for possible upslope movements. Another opportunity relates to co-opting management strategies that are more practically developed and implemented in response to some other issue of management concern. In the face of climate change, limber pine management may require efforts to ensure enough individuals establish in diverse land facets or microclimates in order to increase the odds of some persisting as refugia and future seed sources. A very similar management strategy has been advanced in response to white pine blister rust, where the enrichment planting of seedlings from rust resistant parents is considered a potential approach to reducing vulnerability [71]. Hence, implementation of this management strategy to reduce limber pine vulnerability to the rust pathogen would have the added benefit of reducing vulnerability to climate change. Two possible modifications would need to be considered: (i) ensuring a planting coverage of heterogeneous environments that may serve as future climate refugia, and (ii) adoption of seed sources that – in addition to exhibiting rust resistance – have known physiological responses to climate change [25]. This co-opting of management strategies is also noteworthy in that it allows climate change and rust impacts to be jointly managed without having to explicitly forecast their complex yet weakly understood interactions. Ultimately, the value judgments that direct management actions at Rocky Mountain National Park remain to be seen. The modeling presented here offers significant insights into future scenario planning efforts and will assist in dialog necessary to reconcile disparate social values related to active management and intervention in a national park that is 95% designated wilderness.

Finally, stepping back from a focus on limber pine and Rocky Mountain National Park, the conceptual and analytical framework presented here affords new opportunities to evaluate the potential impacts (i.e., exposure×sensitivity [29]) of future climate change on species' distributions at management-relevant scales. Although the use of correlative distribution models to quantify such impacts is not new, an important advancement for management is the ability to explicitly quantify uncertainty introduced by not knowing the spatial extent at which species' climatic sensitivities should be inferred and used to project responses to climate change. In this context, the framework for better linking correlative species distribution models to management needs benefits in full from all of the various modeling methods available [41]–[43]. Applications in other species and geographies have the potential to encourage the use of correlative distribution models in developing 21^st^ century management scenarios for protected areas, LCCs, and other landscape-scale partnerships.

## References

[1] Scott JM, Davis F, Csuti B, Noss RF, Butterfield B, et al (1993) Gap analysis: a geographic approach to protection of biological diversity. Wildlife Monogr 123: 3–41.

[2] Soulé ME, Terborgh J, editors (1999) Continental Conservation: Scientific Foundations of Regional Reserve Networks. Washington DC: Island Press.

[3] Rodrigues ASL, Andelman SJ, Bakarr MI, Boitani L, Brooks TM, et al (2004) Effectiveness of the global protected area network in representing species diversity. Nature 428: 640–643. 15071592 10.1038/nature02422

[4] Hannah L, Midgley GF, Lovejoy T, Bond WJ, Bush M, et al (2002) Conservation of biodiversity in a changing climate. Conserv Biol 16: 264–268. 10.1046/j.1523-1739.2002.00465.x 35701969

[5] Araújo MB, Cabeza M, Thuiller W, Hannah L, Williams PH (2004) Would climate change drive species out of reserves? An assessment of existing reserve-selection methods. Glob Chang Biol 10: 1618–1626.

[6] Hannah L, Midgley G, Andelman S, Araújo M, Hughes G, et al (2007) Protected area needs in a changing climate. Front Ecol Environ 5: 131–138.

[7] The Secretary of the Interior (2009) Order Number 3289: Assessing the Impacts of Climate Change on America's Water, Land, and other Natural and Cultural Resources. Washington DC.

[8] Cortner HJ, Moote MA (1994) Trends and issues in land and water resources management: Setting the agenda for change. Environ Manage 18: 167–173.

[9] Cheever F (1997) The United States Forest Service and National Park Service: Paradoxical mandates, powerful founders, and the rise and fall of agency discretion. Denv U L Rev 74: 625–648.

[10] Rebertus AJ, Burns BR, Veblen TT (1991) Stand dynamics of *Pinus flexilis*-dominated subalpine forests in the Colorado Front Range. J Veg Sci 2: 445–458.

[11] Lanner RM, Vander Wall SB (1980) Dispersal of limber pine seed by Clark's nutcracker. J Forest 78: 637–639.

[12] Benkman CW (1995) The impact of tree squirrels (*Tamiasciurus*) on limber pine seed dispersal adaptations. Evolution 49: 585–592. 28565136 10.1111/j.1558-5646.1995.tb02295.x

[13] McCutchen HE (1996) Limber pine and bears. Great Basin Nat 56: 90–92.

[14] Logan JA, Powell JA (2001) Ghost forests, global warming, and the mountain pine beetle (Coleoptera : Scolytidae). Am Entomol 47: 160–173.

[15] Baumeister D, Callaway RM (2006) Facilitation by *Pinus flexilis* during succession: A hierarchy of mechanisms benefits other plant species. Ecology 87: 1816–1830. 16922330 10.1890/0012-9658(2006)87[1816:fbpfds]2.0.co;2

[16] Schoettle AW (2004) Developing proactive management options to sustain bristlecone and limber pine ecosystems in the presence of a non-native pathogen. USDA Forest Service Proceedings RMRS-P-34. Fort Collins: Rocky Mountain Research Station.

[17] Rocky Mountain National Park (2010) List of Species of Management Concern. National Park Service

[18] Gibson K, Skov K, Kegley S, Jorgensen C, Smith S, et al.. (2008) Mountain pine beetle impacts in high-elevation five-needle pines: Current trends and challenges. Missoula: USDA Forest Service, Forest Health Protection.

[19] Raffa KF, Aukema BH, Bentz BJ, Carroll AL, Hicke JA, et al (2008) Cross-scale drivers of natural disturbance prone to anthropogenic amplification: The dynamics of bark beetle eruptions. BioScience 58: 501–517.

[20] Johnson DW, Jacobi WR (2000) First report of white pine blister rust in Colorado. Plant Dis 84: 595. 10.1094/PDIS.2000.84.5.595D 30841369

[21] Schoettle AW, Sniezko RA (2007) Proactive intervention to sustain high-elevation pine ecosystems threatened by white pine blister rust. J For Res 12: 327–336.

[22] Boland GJ, Melzer MS, Hopkin A, Higgins V, Nassuth A (2004) Climate change and plant diseases in Ontario. Can J Plant Pathol 26: 335–350.

[23] Mitton JB, Ferrenberg SM (2012) Mountain pine beetle develops an unprecedented summer generation in response to climate warming. Am Nat 179: E163–E171. 22504550 10.1086/665007

[24] Millar CI, Westfall RD, Delany DL (2007) Response of high-elevation limber pine (*Pinus flexilis*) to multiyear droughts and 20th-century warming, Sierra Nevada, California, USA. Can J Forest Res 37: 2508–2520.

[25] Reinhardt K, Castanha C, Germino MJ, Kueppers LM (2011) Ecophysiological variation in two provenances of *Pinus flexilis* seedlings across an elevation gradient from forest to alpine. Tree Physiol 31: 615–625. 21757486 10.1093/treephys/tpr055

[26] Moyes AB, Castanha C, Germino MJ, Kueppers LM (2013) Warming and the dependence of limber pine (*Pinus flexilis*) establishment on summer soil moisture within and above its current elevation range. Oecologia 171: 271–282. 22875149 10.1007/s00442-012-2410-0

[27] Schoettle AW, Rochelle SG (2000) Morphological variation of *Pinus flexilis* (Pinaceae), a bird-dispersed pine, across a range of elevations. Am J Bot 87: 1797–1806. 11118417

[28] Little EL Jr (1971) Atlas of United States Trees, Volume 1: Conifers and Important Hardwoods. US Department of Agriculture Miscellaneous Publication 1146.

[29] Glick P, Stein BA, Edelson NA, editors (2011) Scanning the Conservation Horizon: A Guide to Climate Change Vulnerability Assessment. Washington DC: National Wildlife Federation.

[30] Trivedi MR, Berry PM, Morecroft MD, Dawson TP (2008) Spatial scale affects bioclimate model projections of climate change impacts on mountain plants. Glob Chang Biol 14: 1089–1103.

[31] Rogelj J, Meinshausen M, Knutti R (2012) Global warming under old and new scenarios using IPCC climate sensitivity range estimates. Nature Climate Change 2: 248–253.

[32] Lockman IB, DeNitto GA (2007) WLIS: The Whitebark-Limber Pine Information System and What It Can Do for You. R6-NR-FHP-2007-01. Missoula: USDA Forest Service.

[33] Salas DE, Stevens J, Schulz K (2005) USGS-NPS Vegetation Mapping Program: Rocky Mountain National Park, Colorado: 2001–2005 Vegetation Classification and Mapping. Tech. Memorandum 8260-0502. Denver: Bureau of Reclamation.

[34] PRISM Climate Group (2012) PRISM Climate Data. Available from http://www.prism.oregonstate.edu/. Accessed 2013 Nov.

[35] Thrasher B, Xiong J, Wang W, Melton F, Michaelis A, et al (2013) Downscaled climate projections suitable for resource management. Eos Trans AGU 94: 321–323.

[36] Wood AW, Maurer EP, Kumar A, Lettenmaier DP (2002) Long-range experimental hydrologic forecasting for the eastern United States. J Geophys Res 107: 4429.

[37] Wood AW, Leung LR, Sridhar V, Lettenmaier DP (2004) Hydrologic implications of dynamical and statistical approaches to downscaling climate model outputs. Climatic Change 15: 189–216.

[38] Maurer EP, Hidalgo HG (2008) Utility of daily vs. monthly large-scale climate data: An intercomparison of two statistical downscaling methods. Hydrol Earth Syst Sci 12: 551–563.

[39] Hijmans R (2006) Bioclim-aml, version 2.3. Berkeley: Museum of Vertebrate Zoology. Available from http://www.worldclim.org/mkBCvars.aml. Accessed 2013 Nov.

[40] Phillips SJ, Anderson RP, Schapire RE (2006) Maximum entropy modeling of species geographic distributions. Ecol Model 190: 231–259.

[41] Elith J, Graham CH, Anderson RP, Dudík M, Ferrier S, et al (2006) Novel methods improve prediction of species' distributions from occurrence data. Ecography 29: 129–151.

[42] Midgley GF, Davies ID, Albert CH, Altwegg R, Hannah L, et al (2010) BioMove – an integrated platform simulating the dynamic response of species to environmental change. Ecography 33: 612–616.

[43] Schumaker NH (2013) HexSim. US Environmental Protection Agency, Environmental Research Laboratory, Corvallis, Oregon. Available from http://www.hexsim.net. Accessed 2013 Nov.

[44] Seo C, Thorne JH, Hannah L, Thuiller W (2009) Scale effects in species distribution models: Implications for conservation planning under climate change. Biol Lett 5: 39–43. 18986960 10.1098/rsbl.2008.0476 PMC2657743

[45] Commission for Environmental Cooperation (2011) North American Terrestrial Ecoregions—Level III. Commission for Environmental Cooperation: Montréal, Québec. Available from http://www.cec.org. Accessed 2013 Nov.

[46] Phillips SJ, Dudík M (2008) Modeling of species distributions with MaxEnt: New extensions and a comprehensive evaluation. Ecography 31: 161–175.

[47] Warren DL, Glor RE, Turelli M (2010) ENMTools: A toolbox for comparative studies of environmental niche models. Ecography 33: 607–611.

[48] Warren DL, Seifert SN (2011) Environmental niche modeling in MaxEnt: The importance of model complexity and the performance of model selection criteria. Ecol Appl 21: 335–342. 21563566 10.1890/10-1171.1

[49] Burnham KP, Anderson DR (2002) Model Selection and Multimodel Inference: A Practical Information-Theoretic Approach, Second Edition. New York: Springer-Verlag.

[50] Gesch D, Oimoen M, Greenlee S, Nelson C, Steuck M, et al (2002) The National Elevation Dataset. Photogramm Eng Rem S 68: 5–11.

[51] Elith J, Kearney M, Phillips SJ (2010) The art of modeling range-shifting species. Methods Ecol Evol 1: 330–342.

[52] Elith J, Phillips SJ, Hastie T, Dudík M, Chee YE, et al (2011) A statistical explanation of MaxEnt for ecologists. Diversity Distrib 17: 43–57.

[53] Jackson ST, Betancourt JL, Booth RK, Gray ST (2009) Ecology and the ratchet of events: Climate variability, niche dimensions, and species distributions. Proc Natl Acad Sci USA 106: 19685–19692. 19805104 10.1073/pnas.0901644106 PMC2780932

[54] McLaughlin BC, Zavaleta ES (2012) Predicting species responses to climate change: Demography and climate microrefugia in California valley oak (*Quercus lobata*). Glob Chang Biol 18: 2301–2312.

[55] Stohlgren TJ, Owen AJ, Lee M (2000) Monitoring shifts in plant diversity in response to climate change: A method for landscapes. Biodivers Conserv 9: 65–86.

[56] Brown PM, editor (2013) OLDLIST. Fort Collins: Rocky Mountain Tree Ring Research. Available from http://www.rmtrr.org/. Accessed 2013 Nov.

[57] Sibold JS, Veblen TT, González ME (2006) Relationships of subalpine forest fires in the Colorado Front Range with inter-annual and multidecadal-scale climatic variation. J Biogeogr 33: 833–842.

[58] Stohlgren TJ, Bachand RR (1997) Lodgepole pine (*Pinus contorta*) ecotones in Rocky Mountgain National Park, Colorado, USA. Ecology 78: 632–641.

[59] Donnegan JA, Rebertus AJ (1999) Rates and mechanisms of subalpine forest succession along an environmental gradient. Ecology 80: 1370–1384.

[60] Smith CM, Poll G, Gillies C, Praymak C, Miranda E, et al.. (2011) Limber pine seed and seedling planting experiment in Waterton Lakes National Park, Canada. In: Keane RE, Tomback DF, Murray MP, Smith CM, editors. The future of high-elevation white pines in Western North America: Proceedings of the High Five Symposium, RMRS-P-63. Fort Collins: Rocky Mountain Research Station.

[61] Hessl AE, Baker WL (1997) Spruce and fir regeneration and climate in the forest-tundra ecotone of Rocky Mountain National Park, Colorado, USA. Arctic Alpine Res 29: 173–183.

[62] McWethy DB, Gray ST, Higuera PE, Littell JS, Pederson GT, et al.. (2010) Climate and terrestrial ecosystem change in the U.S. Rocky Mountains and Upper Columbia Basin: Historical and future perspectives for natural resource management. Natural Resource Report NPS/GRYN/NRR—2010/260. National Park Service, Fort Collins, Colorado.

[63] Coop JD, Schoettle AW (2009) Regeneration of Rocky Mountain bristlecone pine (*Pinus aristata*) and limber pine (*Pinus flexilis*) three decades after stand replacing fires. Forest Ecol Manag 257: 893–903.

[64] Natural Resources Conservation Service (2006) Soil Survey of Rocky Mountain National Park, Colorado. US Department of Agriculture. Available from http://soils.usda.gov/survey/online_surveys/colorado/. Accessed 2013 Nov.

[65] Anderson RS, Betancourt JL, Mead JI, Hevly RH, Adam DP (2000) Middle- and late-Wisconsin paleobotanic and paleoclimatic records from the southern Colorado Plateau, USA. Palaeogeogr Palaeoclimateol Palaeoecol 155: 31–57.

[66] Means RE (2011) Synthesis of lower treeline limber pine (*Pinus flexilis*) woodland knowledge, research needs, and management considerations. In: Keane RE, Tomback DF, Murray MP, Smith CM, editors. The future of high-elevation white pines in Western North America: Proceedings of the High Five Symposium, RMRS-P-63. Fort Collins: Rocky Mountain Research Station.

[67] Rochefort RM, Little RL, Woodward A, Peterson DL (1994) Changes in sub-alpine tree distribution in western North America: A review of climatic and other causal factors. Holocene 4: 89–100.

[68] Harsch MA, Hulme PE, McGlone MS, Duncan RP (2009) Are treelines advancing? A global meta-analysis of treeline response to climate warming. Ecol Lett 12: 1040–1049. 19682007 10.1111/j.1461-0248.2009.01355.x

[69] Ashton I, Schweiger EW, Burke J, Shorrock D, Pillmore D, et al.. (2010) Alpine vegetation composition structure and soils monitoring protocol: 2010 version. Natural Resource Report NPS/ROMN/NRR—2010/277. Fort Collins: National Park Service.

[70] Ashton I (2011) Alpine vegetation composition, structure, and soils for Rocky Mountain National Park: 2010 summary report. Natural Resource Report NPS/ROMN/NRDS—2011/148. Fort Collins: National Park Service.

[71] Schoettle AW, Goodrich BA, Klutsch JG, Burns KS, Costello S, et al.. (2011) The proactive strategy for sustaining five-needle pine populations: An example of its implementation in the southern Rocky Mountains. In: Keane RE, Tomback DF, Murray MP, Smith CM, editors. The future of high-elevation white pines in Western North America: Proceedings of the High Five Symposium, RMRS-P-63. Fort Collins: Rocky Mountain Research Station.

